# Epigenetic dysregulated long non-coding RNAs in renal cell carcinoma based on multi-omics data and their influence on target drugs sensibility

**DOI:** 10.3389/fgene.2024.1406150

**Published:** 2024-08-02

**Authors:** Jiawei Wang, Pingnan Dou, Yunwen Sun, Jie Zheng, Guanwei Wu, Heqian Liu, Lingsong Tao

**Affiliations:** ^1^ Department of Urology, The Second People’s Hospital of Wuhu, Wuhu, China; ^2^ School of Pediatrics, Nanjing Medical University, Nanjing, China; ^3^ The First Clinical Medical College of Nanjing Medical University, Nanjing, China

**Keywords:** lncRNAs, ccRCC, sensitivity, epigenetic, prognosis

## Abstract

**Purpose:**

Epigenetic modifications play a crucial role in cancer development, and our study utilized public data to analyze which leads to the discovery of significant epigenetic abnormalities in lncRNAs, offering valuable insights into prognosis and treatment strategies for renal carcinoma.

**Methods:**

Public data were obtained from the Cancer Genome Atlas (TCGA), International Cancer Genome Consortium (ICGC) and Gene Expression Omnibus (GEO) database. The analysis of the online public data was all completed in R software.

**Results:**

We discovered a great number of epigenetic abnormalities of lncRNA in renal cancer, which is achieved by comparing the following modification and methylation of histone region changes on the promoter and enhancer of lncRNA: H3K27ac, H3K4me1, H3K4me3. As a result, 12 specific epigenetic disorders of lncRNA genes in renal cancer were identified. Finally, based on this lncRNA, we investigated the prognosis of renal cancer samples, among which 8 lncRNA can be seen as markers of prognosis in renal cancer, which had great prediction ability for ccRCC prognosis. Meanwhile, high risk score may pose response better to axitinib and nilotinib, but not sorafenib or sunitinib. Beyond, we observed an elevated level of risk score in immunotherapy non-responders. Further, biological enrichment and immuno-infiltration analysis was conducted to investigate the fundamental differences between patients categorized as high or low risk.

**Conclusion:**

Our research improves the understanding in the function of epigenetic dysregulated long non-coding RNAs in renal carcinoma.

## 1 Introduction

Renal cell carcinoma is predominantly represented by clear cell renal cell carcinoma (ccRCC), constituting over three-quarter of all reported cases, making it the most significant histological subtype among renal carcinomas (Capitanio and Montorsi, 2016). Early-stage patients with renal cancer who undergo surgery might benefit from long-term therapeutic outcomes (Campbell et al., 2021). Nonetheless, for those with advanced stage or distant metastasis, treatment options are often limited. Consequently, identifying novel targets that have the potential to serve as ones of guiding renal carcinoma therapy is meaningful.

Epigenetic modification is a common physiological process in organisms, like DNA methylation, genomic imprinting, gene silencing, and so on (Miranda Furtado et al., 2019). Generally, normal and orderly epigenetics help maintain normal physiological functions of cells and the body. However, there is increasing evidence that epigenetic regulation abnormalities are closely linked to cancer occurrence and development (Sun et al., 2022). Becker et al. (2020) found that epigenetic reprogramming can maintain the glycolytic phenotype of breast carcinoma cells by regulating HIF-1α and glycolytic enzymes, further enhancing cancer progression. Hamamoto et al. (2004) revealed that SMYD3 was overexpressed in colon and liver cancer, and can facilitate cancer development based on its histone methyltransferase activity. Meanwhile, Ulanovskaya et al. (2013) indicated that the nicotinamide N-methyltransferase NNMT can affect tumor metabolism by impairing the methylation potential of cancer cells. Long non-coding RNAs (lncRNAs) represent a category of noncoding RNAs that exceed 200 nucleotides in length, famous for their broad regulatory effect (Bhan et al., 2017). Moreover, lncRNAs are widely known of being involved in cancer epigenetics. For example, in colonic carcinoma, Ni et al. (2019) noticed that lncRNA GAS5 has direct interaction with WW, subsequently facilitating the phosphorylation and ubiquitin-mediated degradation of YAP. Xiu et al. (2019) demonstrated that the lncRNA LINC02273 can form a complex with hnRNPL. This complex can upregulate AGR2 transcription by influencing epigenetic modification of the AGR2 promoter region and therefore promote cancer development. Therefore, exploring the lncRNAs involved in epigenetic modification is important.

In this research, we identified the lncRNAs involved in epigenetic modification in ccRCC and comprehensively explored their role. Moreover, based on the identified epi-lncRNAs, a prognosis model was established with good prediction ability of patients’ prognosis. In addition, drug-susceptibility analysis illustrated that patients who got high-risk scores may have a more positive response to axitinib and nilotinib than to sorafenib or sunitinib. Meanwhile, we noticed a higher risk score among non-responders with immunotherapy. Further, biological enrichment and immuno-infiltration analysis was published to investigate the fundamental differences between patients categorized as high or low risk. Our result can provide novel insight into the identified epi-lncRNAs.

## 2 Materials and methods

### 2.1 Public data collection

In the research, the gene expression profile of ccRCC and its corresponding normal samples were downloaded from International Cancer Genome Consortium (ICGC) and Cancer Genome Atlas (TCGA) database, including FPKM, count number expression profile data and clinical information. Then we converted FPKM to TPM based on the author’s R code. GENCODE’s gene annotation file (version 24) was used to differentiate between long noncoding RNAs and protein-coding genes (PCGs). We also transformed the Ensembl ID of the chosen genes into symbol form. All expression profiles have been processed before analysis. Also, 450k microarray chip data were downloaded from the TCGA-KIRC project. Besides, all the missing values in renal cancer data were completed with the KNN method. Based on the Gene Expression Omnibus (GEO) database, the GSE86091 dataset with para-cancerous and tumor samples was downloaded, including h3k4me1, h3k4me3, h3k27ac histone information (Yao et al., 2017).

### 2.2 Identification of lncRNAs and PCGs associated with the epigenetic disorder

Then, the lncRNAs and PCGs which expressed differentially were identified by R package deseq2 with *p*-value determined by the Benjamin Hochberg method. LncRNAs or PCGs with FDR < 0.05 and |logFC| > 1 were considered significant. Next, we selected the unique peak in RCC in accordance with the physical location of the histone modified peak. Only the peak with a *p*-value < 0.05 was retained as the difference peak. Furthermore, we combined with the GTF file of GENCODE to get differentially histone-modified genes as well as identity of gene enhancers from the human enhancer database FANTOM5. The promoter of the gene is defined as 2 kb upstream and 0.5 kb downstream of the transcription initiation site (TSS), which can be identified by the R package ChIPseeker and the human lincRNAsTranscripts database. Finally, Using R-packet CHAMP, the Bumphunter method is used to identify DMR and the regions with Bumphunter DMR. *p*-values < 0.01 are considered significant DMRs. In the end, we made a definition that lncRNAs meet the following conditions as epiRNAs: i) there was differential expression between tumor and normal samples; ii) promoter or enhancer has more than one histone modified or methylated region.

### 2.3 Genomic characterization

To figure out the genomic characteristics of epigenetic dysregulation and none dysregulation lncRNA/PCG, we made comparations among the number and length of exons, transcripts, genes of epi-lncRNA, non-epi-lncRNA, epi-PCG, non-epi-PCG.

### 2.4 Genome mapping

To investigate the epigenetic features of lncRNA caused by histone modification, we analyzed the distribution characteristics of promoter and enhancer among epi-lncRNA modified through different histones in genome level.

### 2.5 Identification of LncRNA with renal carcinoma-specific candidate epigenetic characteristic

To learn more about the relation between epigenetic disorders lncRNA and carcinoma, we made an analysis of epi-lncRNA enrichment and cancer lncRNAs with lnc2cancer v3.0. Besides, epi-lncRNA related to ccRCC was screened out for the sake of exploring the regulation of epi-lncRNA. In addition, considering that most of the genes that affect the process of the diseases tend to show expression disorder, we identified the epi-lncRNA in ccRCC through the TCGA TPM expression profile.

### 2.6 Enrichment analysis

Pathway enrichment and quantification of specific pathways activity were completed by Gene Set Enrichment Analysis (GSEA) and Single Sample GSEA (ssGSEA) algorithm, including the Kyoto Encyclopedia of Genes and Genomes (KEGG) and epigenetically dysregulated lncRNAs score (Subramanian et al., 2005; Pang Y et al., 2022).

### 2.7 Prognosis analysis

The Kaplan-Meier method was applied to estimate the overall survival (OS) rate and survival time, and based on the method of log-rank test, the differences among different sample subgroups were obtained. Finally, the potential prognostic markers of epi-lncRNA were identified. A risk model was built on basis of multivariate Cox regression analysis.

### 2.8 Immune infiltration analysis

The CIBERSORT algorithm (version 1.03) and the LM22 signature matrix were used to analyze immune infiltration according to the expression profile (Chen et al., 2018).

### 2.9 Drug sensitivity and immunotherapy analysis

The Genomics of Drug Sensitivity in Cancer Database (GDSC) was used to analyze drug susceptibility (Yang et al., 2013). The evaluation of patients taken immunotherapy was made by application of the Tumor Immune Dysfunction and Exclusion (TIDE) algorithm (Fu et al., 2020).

### 2.10 Statistical analysis

All analysis was performed with the programs of R software (version 4.2.1). Statistical significance was determined using two-tailed *p*-value and less than 0.05 was considered statistically significant.

### 2.11 Clinical samples and tissue

This study was approved by the Institutional Review Board of the First Affiliated Hospital of Nanjing Medical University. All patients participated in the study voluntarily donated samples for clinical research and signed informed consents before surgery. We collected samples from patients with renal cell carcinoma from March 2024 to June 2024. All the samples were from patients took partial nephrectomy in the Department of Urology Department of the First Affiliated Hospital of Nanjing Medical University. Samples were collected under the guidance of one experienced pathologist, and immediately stored in a liquid nitrogen tank.

### 2.12 RNA preparation and quantitative real-time PCR (qRT-PCR)

TRIZOL reagent (Invitrogen) was used to extract total RNA in both tissues and cells according to the manufacturer’s instruction. The cDNA was obtained from the purified RNA using a PrimeScript RT Reagent Kit (Takara). SYBR Premix Ex Taq II Kit (Takara) was used for qRT-PCR assays. Results were normalized to β-actin expression. All experiments were performed at least three times. All specific primers used in this study have been listed in Supplementary Table S1.

## 3 Results

### 3.1 Identification of the epigenetically dysregulated lncRNAs

**FIGURE 1:**
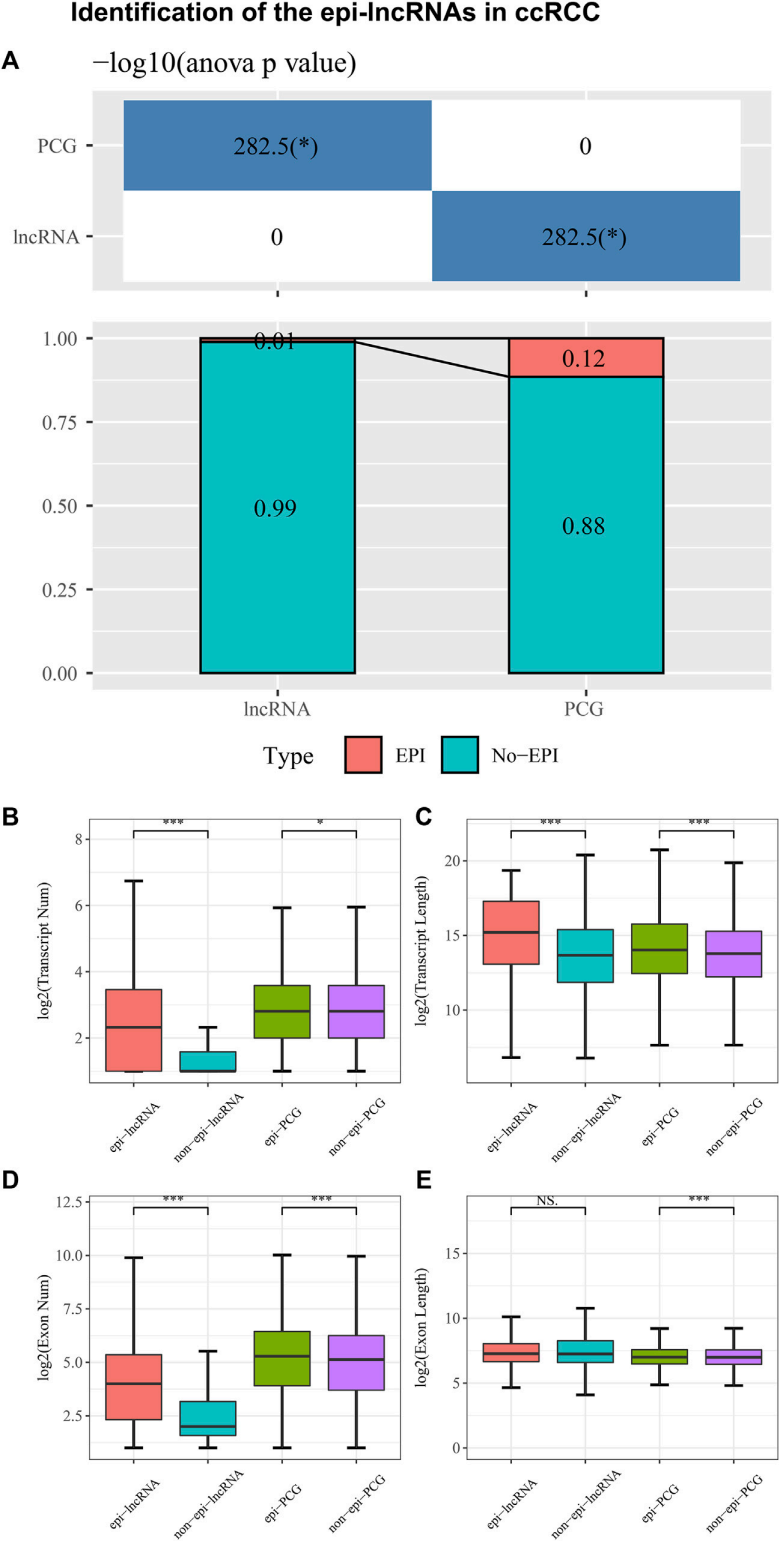
Identification of the epi-lncRNAs in ccRCC. Notes: **(A)** The proportion of epi-lncRNAs and epi-PCGs to total lncRNAs and PCGs in the genome; **(B–E)** Genomic characteristics comparison between epigenetic dysregulated lncRNA/PCGs and non-epigenetic dysregulated lncRNA/PCGs.

Genes that are differentially expressed were identified using DESeq2, including 5906 PCGs and 5370 lncRNAs. The combination of histone modifications and 450K methylation microarray data enabled us to identify 149 epiRNAs, 13426 non-epiRNAs, 2241 epi-PCGs, and 17200 non-epi-PCGs. Moreover, lncRNAs in ccRCC are significantly less frequently than in PCG (Figure 1A). A comparison of epi-lncRNA transcripts and exons with non-epi-lncRNA transcripts, epi-PCG transcripts, and non-epi-PCG transcripts was followed to represent the genomic characteristics of epigenetically dysregulated lncRNAs. Epi-lncRNAs appear with a long transcript length and large numbers of transcripts (Figures 1B, C) and epi-PCG showed similar structural characteristics as epi-lncRNAs. Simultaneously, epi-lncRNAs were presented with more exons and epi-PCG owns more exons and length (Figures 1D, E).

### 3.2 Genomic landscape of epigenetically dysregulated lncRNAs

**FIGURE 2:**
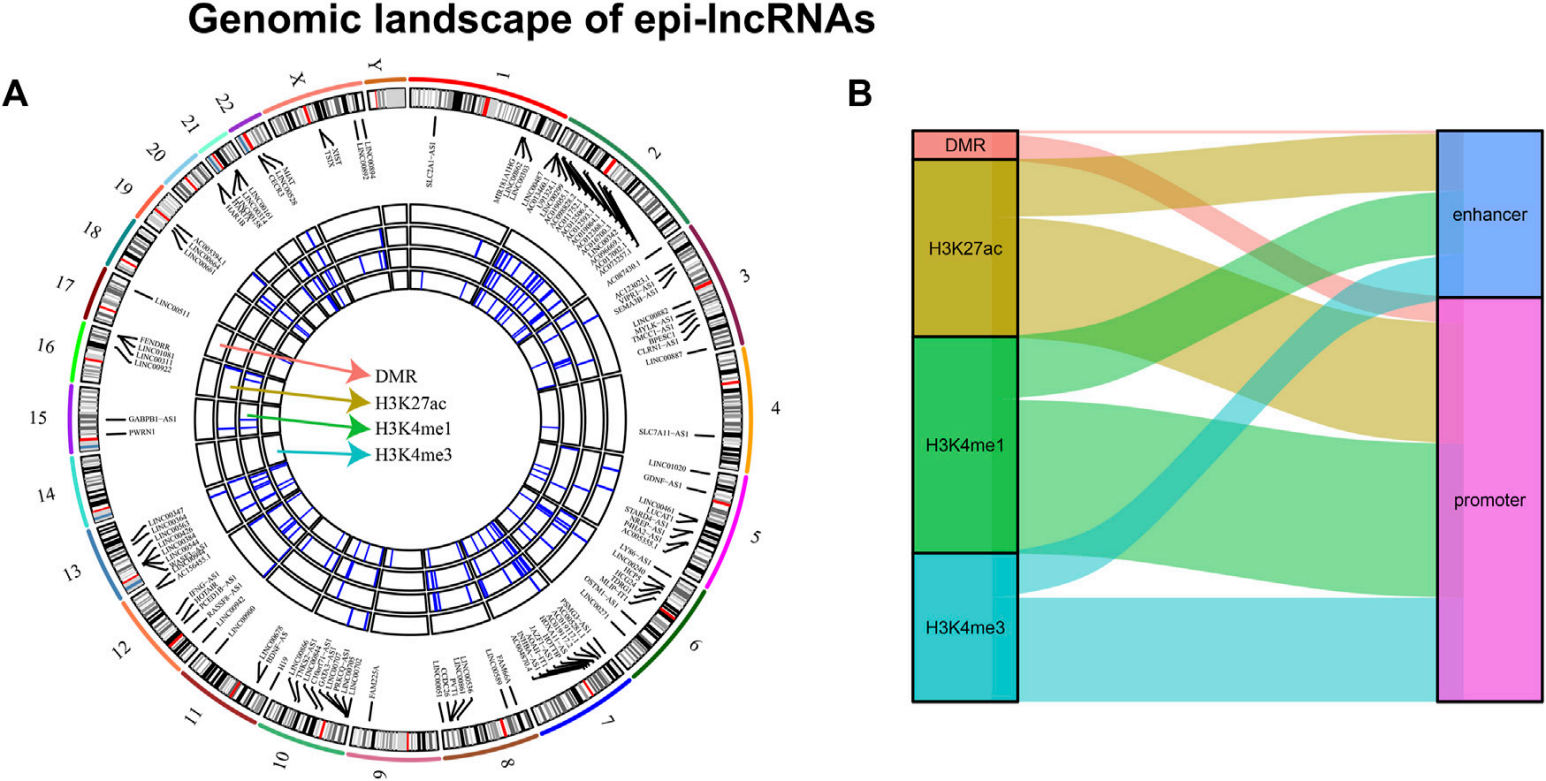
Genomic landscape of epi-lncRNAs. Notes: **(A)** Differential methylation regions and histone modifications lead to a distinct epi-lncRNA genomic landscape; **(B)** Type distribution in epi-lncRNAs with several apparent disorders.

Systematic analysis of epigenetic disorders of epi-lncRNA in renal cell carcinoma revealed the landscape of epi-lncRNAs resulted from various histone modifications and different methylation regions (Figure 2A). Besides, in these lncRNAs, we found several abnormal histone modifications including H3K4me3, H3K4me1, and H3K27ac. Also, most of the lncRNAs with apparent disorders coexist with various histone modifications. Moreover, we also found that the abnormal histone modifications were mostly located in the promoter region (Figure 2B).

### 3.3 Biological enrichment of dysregulated epi-lncRNAs

**FIGURE 3:**
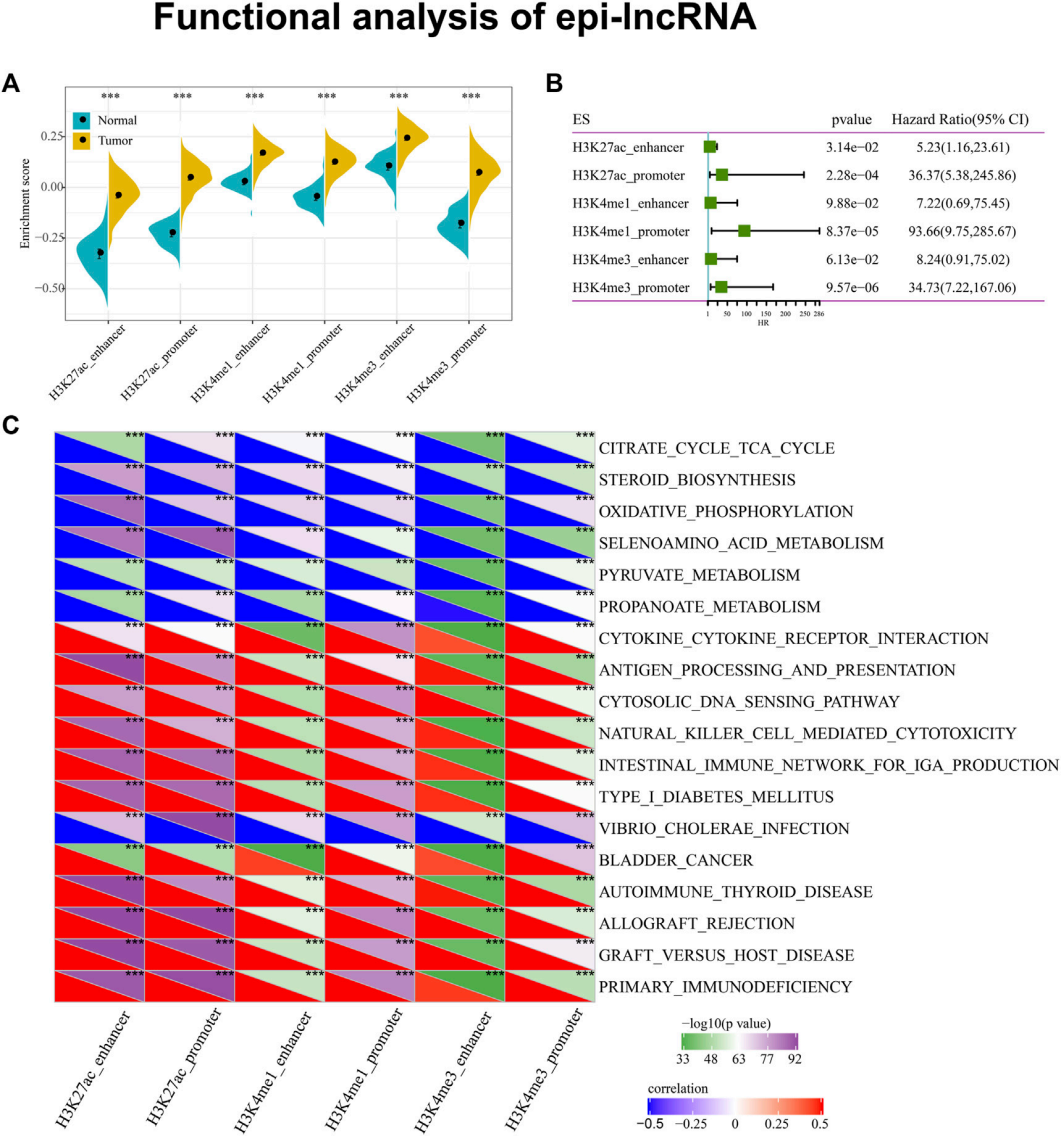
Functional analysis of epi-lncRNA. Notes: **(A)** Differences between cancer and adjacent cancers in six types of apparent dysregulated lncRNAs; **(B)** Relationship between different histone modifications of six epi-lncRNAs and overall survival; **(C)** Six kinds of apparent dysregulated lncRNAs are most related to KEGG pathway.

Next, we completed an analysis of the relation between the expression of epi-lncRNA and the pathway in ccRCC. The ssGSEA was applied to calculate each sample enrichment score in all the lncRNAs affected by histone modifications. We observed that the scores in cancer samples were remarkably higher than adjacent samples, suggesting that these epi-lncRNAs may own a great possibility of being carcinogenic (Figure 3A). Further, we analyzed the relationship between these enrichment scores and OS and found that high scores were associated with poor prognosis (Figure 3B). Additionally, we observed that there are 18 pathways in the KEGG pathway related to six sorts of epi-lncRNA scores closely, suggesting that there is a certain consistency among various epi-lncRNA-related pathways. Of these 18 pathways, the tumor-related pathway of BLADDER_CANCER and the immune-related pathways of PRIMARY_IMMUNODEFICIENCY, CYTOKINE_CYTOKINE_RECEPTOR_INTERACTION, NATURAL_KILLER_CELL_MEDIATED_CYTOTOXICITY and AUTOIMMUNE_THYROID_DISEASE have Positive correlations to the epi-lncRNAs score. On the contrary, the metabolism-related pathways of SELENOAMINO_ACID_METABOLISM, CITRATE_CYCLE_TCA_CYCLE, PYRUVATE_METABOLISM and PROPANOATE_METABOLISM are negatively correlated with the epi-lncRNAs score (Figure 3C). These results reveal that epi-lncRNA plays an important role in tumor development and immunity.

### 3.4 Relationship between epigenetically dysregulated lncRNA and RNA modification

**FIGURE 4:**
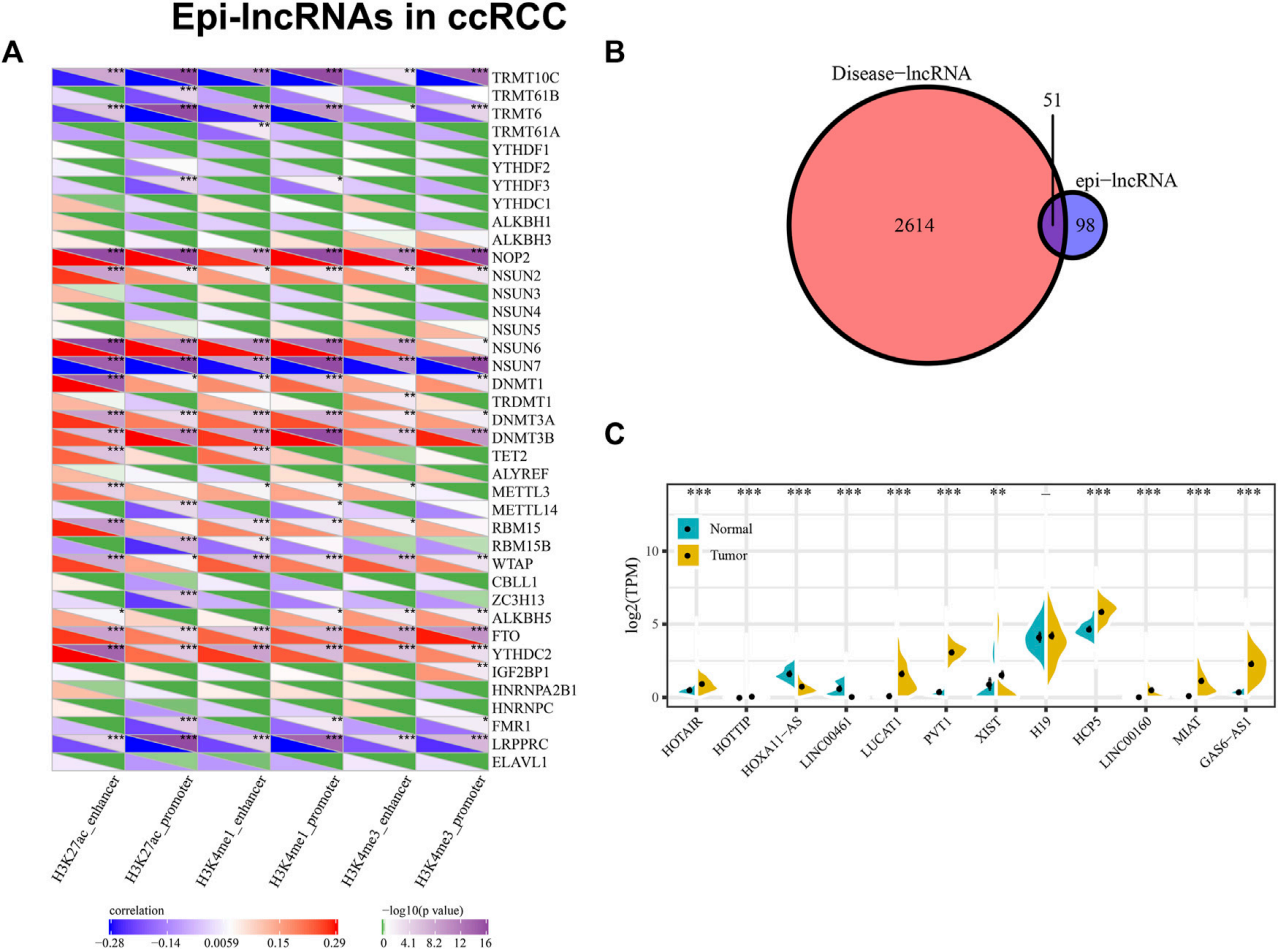
Epi-lncRNAs in ccRCC. Notes: **(A)** Correlation between m6a, m5c, and m1A genes and enrichment scores of six epi-lncRNAs; **(B)** Intersection of disease-related lncRNA and renal cell carcinoma epi-lncRNA; **(C)** Specific epi-lncRNA expression associated with renal cell carcinoma is different between normal samples and tumors.

We calculated the association of the enrichment scores of six epi-lncRNAs and m6a, m5c and m1A genes, indicating that they are significantly related. The enhancer and promoter of H3K4me3, H3K4me1 and H3K27ac not only have a similar correlation with these genes but also have their unique correlation (Figure 4A). The results indicate that there might exist unknown regulatory modes of epi-lncRNA resulted from enhancer and promoter histone modification, with a tight link between epi-lncRNA and RNA modification.

### 3.5 Identification of renal cancer-specific epigenetic dysregulated lncRNA

**FIGURE 5:**
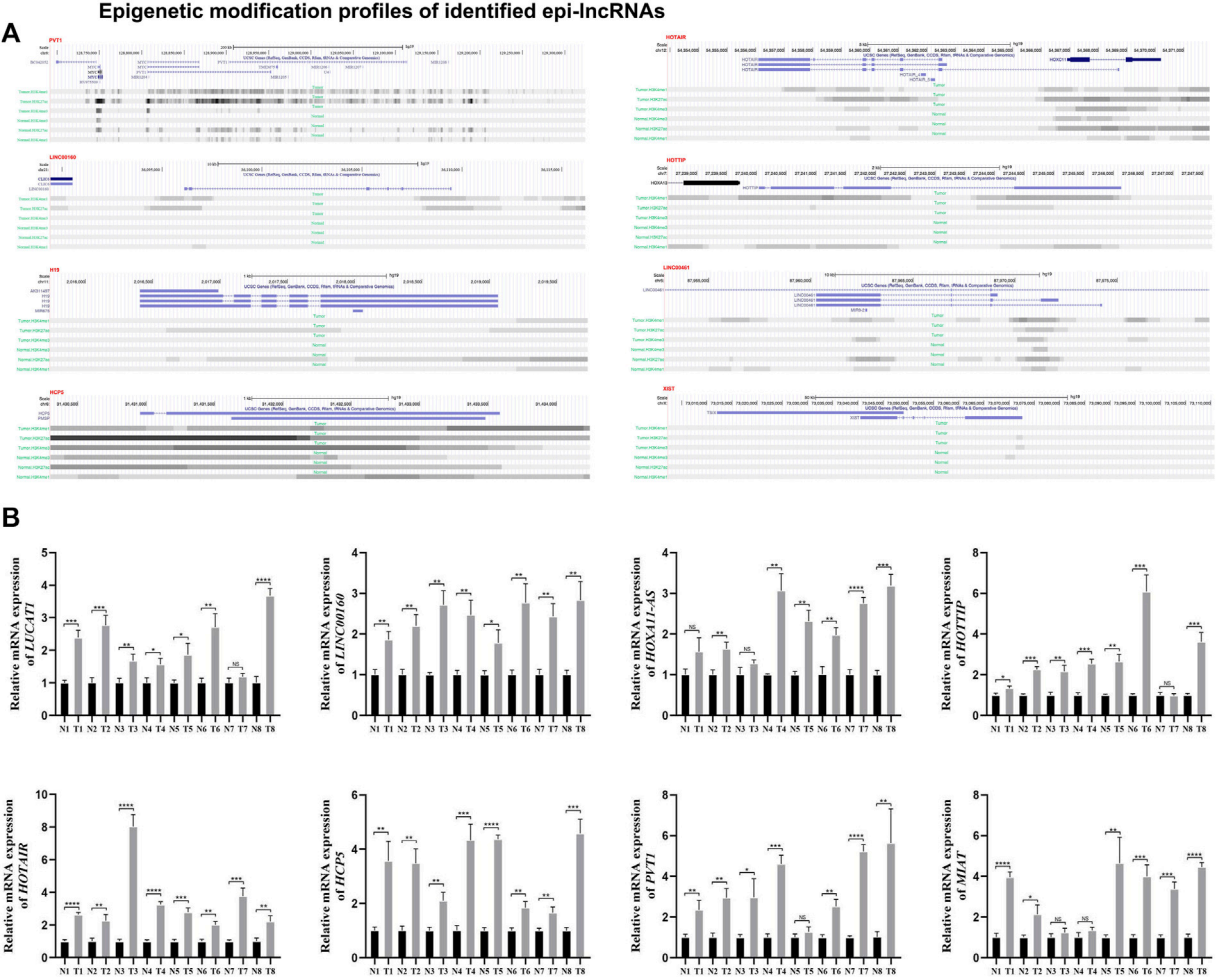
Epigenetic modification profiles of identified epi-lncRNAs. Notes: **(A)** Epigenetic modification profiles of PVT1, HOTAIR, LINC00160, HOTTIP, H19, LINC00461, HCP5 and XIST, **(B)** quantitative real-time PCR validation.

Using Lnc2Cancer V3.0, it can be discovered that 51 epi-lncRNAs were associated with normal human cancers (Figure 4B). To explore how candidate lncRNAs play the regulatory role in ccRCC, the list of ccRCC-related lncRNAs was collected from Lnc2Cancer v3.0. Of 51 epi-lncRNAs, 12 lncRNAs were identified that have direct relationships with ccRCC. We subsequently calculated the differences of 12 epi-lncRNAs between ccRCC samples and normal samples, respectively (Figure 4C). Of that, 11 epi-lncRNAs are presented with significant differences, except H19. Except that the expression of HOXA11-AS and LINC00461 in normal samples is higher than that in tumor samples, the other 9 lncRNAs are highly expressed in tumor samples. Among these epi-lncRNAs, HOTAIR, HOTTIP, XIST, H19, HCP5, and LINC00160 were only related to histone-modified promoters; LINC00461 is only related to DMR enhancers; PVT1 is associated with histone modified enhancer and promoter (Figure 5A). Then, we collected a clinical ccRCC cohrt (normal group: 8, disease group: 8) and performed PCR validation on the relevant lncRNAs, and found significant differences between the disease group and the normal group (Figure 5B).

### 3.6 Epi-lncRNAs are associated with the prognosis of ccRCC

**FIGURE 6:**
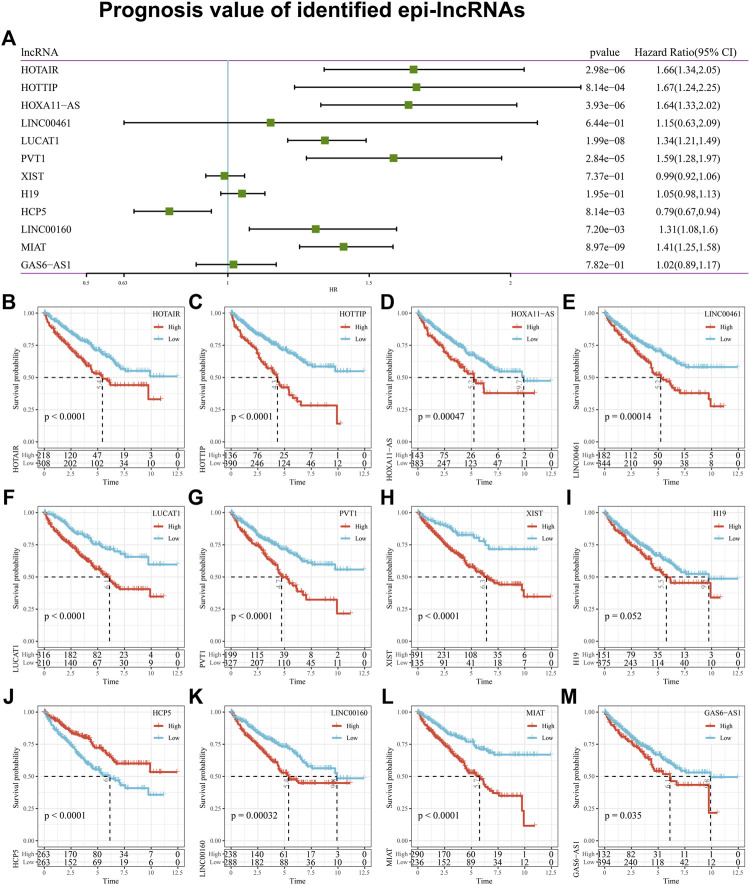
Prognosis value of identified epi-lncRNAs. Notes: **(A)** Univariate analysis of 12 kidney cancer-associated epi-lncRNA genes; **(B–M)** Kaplan-Meier survival curve for samples with high and low expression of 12 renal cell carcinoma-related EPI lncRNA genes.

Considering the potential prognostic value of epi-lncRNA is unclear yet, an analysis of the 12 epi-lncRNAs survival mentioned above based on the sample in TCGA-KIRC was made. Results indicated that 8 epi-lncRNAs are significantly associated with survival based on the univariate Cox regression analysis (Figure 6A). Kaplan-Meier survival curves illustrated that the epi-lncRNAs HOTAIR, HOTTIP, HOXA11-AS1, LINC00461, LUCAT1, PVT1, XIST, H19, LINC00160, MIAT, GAS6-AS1 were correlated with poor prognosis, while HCP5 was correlated with better prognosis (Figures 6B–M).

### 3.7 Establishment of prognosis signature consisting of eight epi-lncRNAs

**FIGURE 7:**
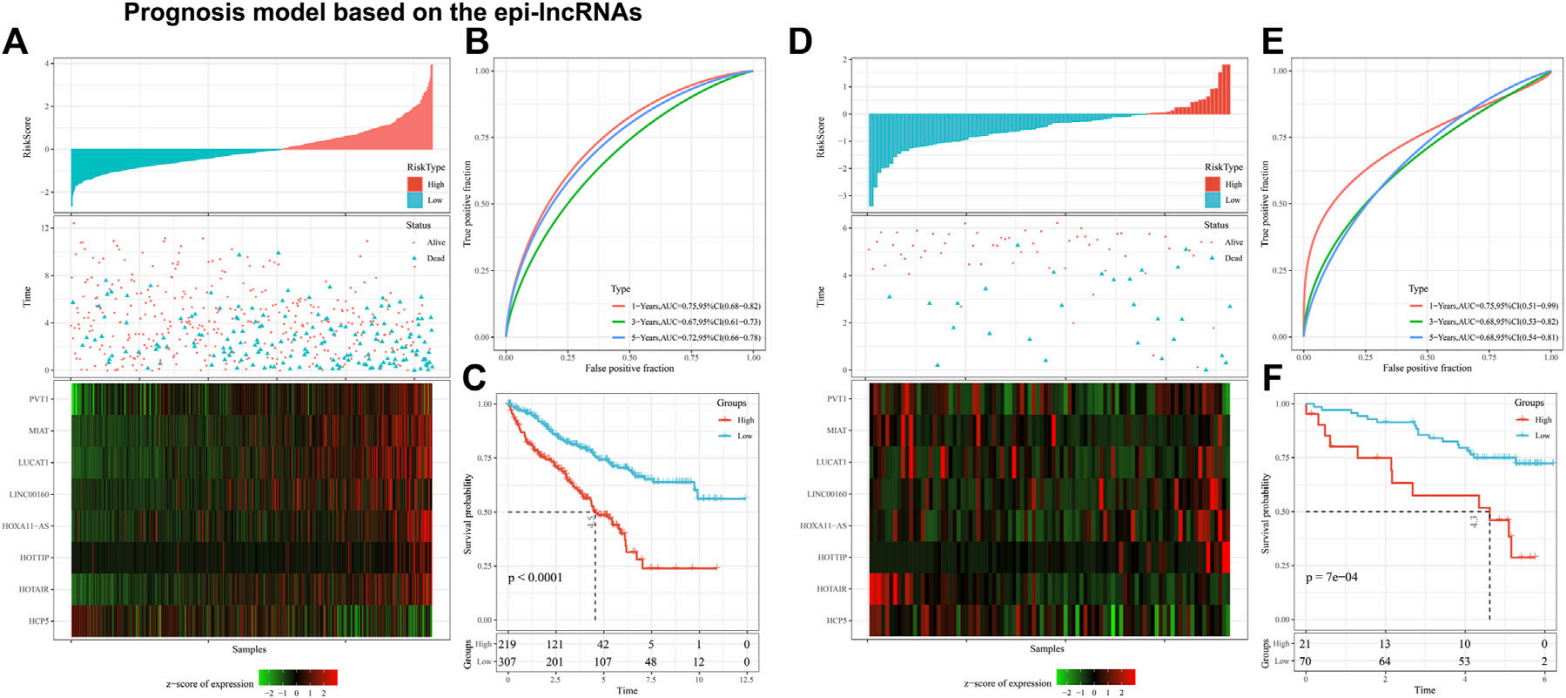
Prognosis model based on the epi-lncRNAs. Notes: **(A)** Overview of our model in TCGA cohort; **(B)** Kaplan-Meier survival curve of high and low risk patients in TCGA cohort; **(C)** ROC curves of our model in TCGA cohort; **(D)** Overview of our model in ICGC cohort; **(E)** Kaplan-Meier survival curve for ICGC cohort patients at high and low risk; **(F)** ROC curves of our model in ICGC cohort.

Multivariate survival analysis was used to establish the 8-EpiLncRNA signature on basis of the TCGA dataset, in which patients with high-risk score had higher mortality (Figure 7A). Receiver operating characteristic curve (ROC) analysis shows that the designed signature owned a remarkable prediction ability of patient OS (Figure 7B, 1-year AUC of 0.75, 3-year AUC of 0.67, and 5-year AUC of 0.72). Similarly, Kaplan-Meier survival curves indicated that the high-risk score patients have a worse OS performance tendency (Figure 7C). Then, we selected the RECA-EU with prognostic information from the ICGC database for model validation. In the ICGC cohort, we also observed satisfactory performance (Figure 7D). The ROC result of 1-, 3- and 5- years were 0.75, 0.68, and 0.68, respectively (Figure 7E). Kaplan-Meier survival curves indicated that the high-risk score ones also incline to have poor prognosis (Figure 7F). Univariate and multivariate analysis suggested that the new model we built can be considered as an independent risk factor of other clinical features (Supplementary Figures S1A, B).

### 3.8 Drug sensitivity and immunotherapy analysis

**FIGURE 8:**
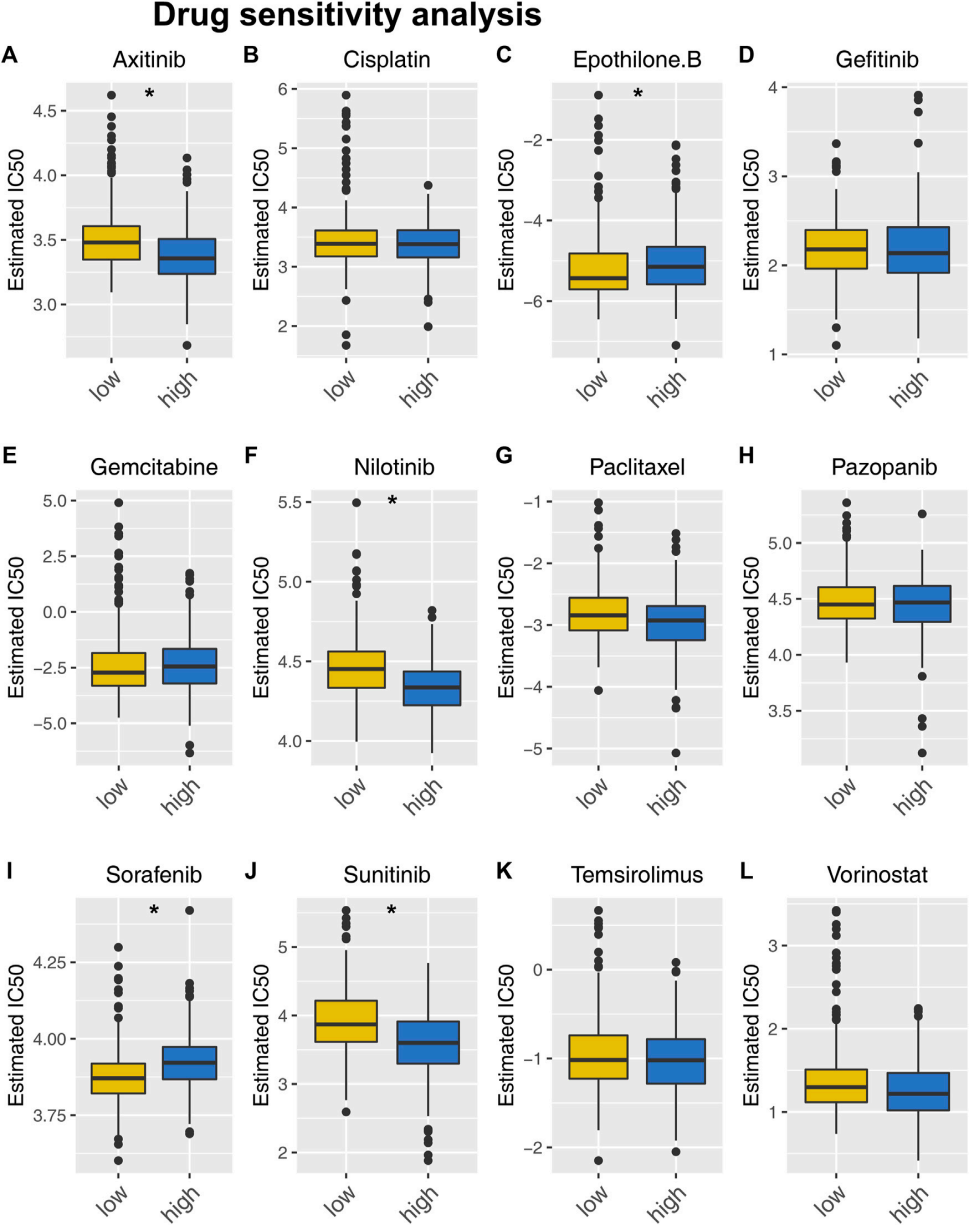
Drug sensitivity analysis. Notes: **(A–L)** Drug sensitivity differences between people at high and low risk for specific target drugs.

Next, we investigated the fundamental distinction in Supplementary Figures S1A, B drug sensitivity between patients at high and low risk. Results indicated patients with high-risk score might respond better to axitinib and nilotinib, but not sorafenib and sunitinib (Figure 8). Moreover, A positive association was found between the TIDE score and the risk score. (Supplementary Figure S2A, cor = 0.274, *p* < 0.001). Meanwhile, it was worth noted that immunotherapy non-responders had a much higher score (Supplementary Figure S2B).

### 3.9 Pathway enrichment and immune infiltration

Based on the Hallmark gene collection, GSEA analysis revealed that the pathways of epithelial-mesenchymal transition, P53 pathway, UV response up, E2F targets, myogenesis, pancreas beta cells, and G2M checkpoint were triggered in patients at high risk (Supplementary Figure S3). Analysis of immune infiltration revealed that there’s a chance that the high-risk patients have more plasma cell infiltration, activated CD4^+^ memory T cells, M0 macrophages, follicular helper T cells, and regulatory T cells (Tregs), while a lower level of monocytes, resting mast cells and eosinophils (Supplementary Figure S4).

## 4 Discussion

Among the most dangerous tumors of the urinary system, renal cancer poses a long-term medical and health burden for patients (Turajlic et al., 2018). Radiotherapy and chemotherapy are ineffective in treating patients with advanced ccRCC patients, therefore limiting their treatment options to some extent (Turajlic et al., 2018). Nowadays, researchers had focused on exploring the target affecting the response of ccRCC patients to specific target drugs. Wei et al. (2021) suggested that MX2 could be a prognosis marker of ccRCC and can affect sunitinib sensibility.

The regulatory effect of lncRNAs has made them possible targets for intervention in cancer. As well as participating in many important physiological processes, lncRNA also contributes to cell remodeling. Tan et al. (2021) found that the posttranslational modifications medicated by lncRNAs can affect the metabolism reprogramming of cancer. Numerous studies have identified dysregulated lncRNAs in RCC tissues compared to normal kidney tissues. These lncRNAs participate in diverse biological processes such as cell proliferation, apoptosis, invasion, and metastasis by modulating chromatin remodeling, transcriptional regulation, and post-transcriptional gene expression. For example, Liu et al. (2022) found that AC010973.2 can affect tumor stemness and is associated with ccRCC patients’ prognosis. Qu et al. (2016) indicated that the exosome-transmitted lncRNA lncARSR can upregulate the level of c-MET and AXL expression in RCC cells through a competitive endogenous RNA (ceRNA) mechanism, partly responsible for the cancer progression and sunitinib resistance. In terms of epigenetic modification, lncRNAs exert their regulatory functions through interactions with epigenetic modifiers including DNA methyltransferases, histone methyltransferases, and chromatin remodeling complexes. They can act as scaffolds, guides, or decoys to target specific genomic loci and regulate gene expression in cis or trans (Gu et al., 2021; Hu et al., 2021; Yang et al., 2021). In conclusion, while significant strides have been made in elucidating the epigenetic landscape of RCC, challenges such as tumor heterogeneity and technical limitations necessitate ongoing research efforts. Future directions should focus on overcoming these challenges through innovative research methodologies and collaborative multiomics and single-cell approaches, ultimately translating scientific discoveries into clinical benefits for RCC patients.

In our research, we discovered the lncRNAs involved in the epigenetic modification in ccRCC. A total of 51 lncRNAs were identified associated with the enriched score of promoter or enhancer of H3K27ac, H3K4me1 and H3K4me3. Among these lncRNAs, 12 epi-lncRNAs were selected for further analysis for their role in ccRCC previously reported, including HOTAIR, HOTTIP, HOXA11-AS, LINC00461, LUCAT1, PVT1, XIST, H19, HCP5, LINC00160, MIAT and GAS6-AS1. In renal cancer, Bai et al. (2021) found that HOTAIR can coordinate with the androgen receptor to enhance the transcription of GLI2, further promoting tumor stemness and angiogenesis. Wang et al. (2018) uncovered that the lncRNA HOTTIP may accelerate the development of renal cancer through a ceRNA mechanism (miR-615/IGF-2 axis). According to reports, all these lncRNAs play a role in renal cancer development. Our result indicated that these lncRNAs were associated with abnormal epigenetic modifications, which can broaden the research direction of these lncRNAs.

Also, based on the identified epi-lncRNAs, a prognosis model was established with good prediction ability of patients’ prognosis. Moreover, the results of drug sensitivity analysis showed that Axitinib and nilotinib may be far more effective for patients with high-risk scores, but not sunitinib and sorafenib. When treating advanced renal cancer, sunitinib is a highly potent targeted drug (Powles et al., 2020). Researchers have focused on the factors affecting the target drug of ccRCC. Xiong et al. (2021) demonstrated how RRM2 can influence PD-1 blockade and sunitinib sensitivity by promoting the AKT pathway and stabilizing ANXA1. Based on our results, assessing the model molecules’ relative expression level might reveal the patient risk group, further guiding the therapy option for renal cancer.

Some shortcomings also need to be addressed. Initially, the patients we registered were mainly Western people. The race bias brought by this can reduce the effectiveness of our findings. Also, the sample counts in the validation cohort ICGC are less than in the TCGA cohort. In the future, advances in high-throughput sequencing technologies and computational algorithms will facilitate integrated analyses of lncRNA expression profiles, epigenetic modifications, and genomic alterations in urologic tumors (Pang et al., 2020; Pang K et al., 2022). This holistic approach promises to unravel complex regulatory networks and identify actionable targets for precision medicine. Moreover, Continued efforts in RNA biology and nanomedicine hold promise for developing innovative RNA-targeted therapies, including lncRNA-specific inhibitors or mimics, for personalized treatment strategies in RCC.

## Data Availability

All the data used for this study was open-accessed through the following link: https://portal.gdc.cancer.gov/ (TCGA databse); https://icgc.org/ (ICGC databse); https://www.ncbi.nlm.nih.gov/geo/query/acc.cgi?acc=GSE86091 (GSE86091). All original data can be obtained from the corresponding author based on reasonable requirements.
