# Supplementary figures and images for: Epigenetic dysregulated long non-coding RNAs in renal cell carcinoma based on multi-omics data and their influence on target drugs sensibility
Source: Front Genet. 2024 Aug 2;15:1406150. doi: 10.3389/fgene.2024.1406150 (PMC11327069; doi:10.3389/fgene.2024.1406150)

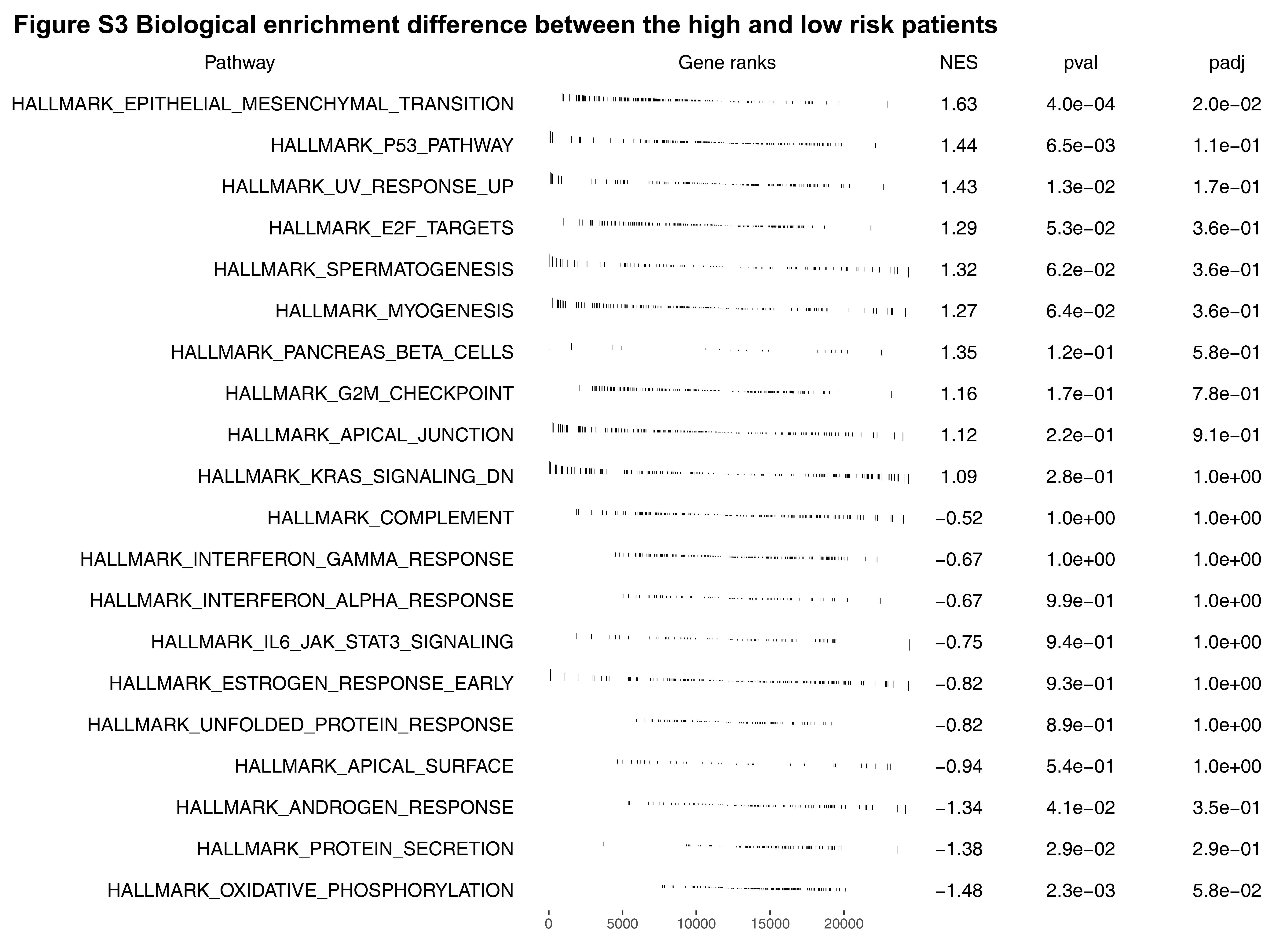

Supplement: Supplementary file 1 [file Image3.TIF]

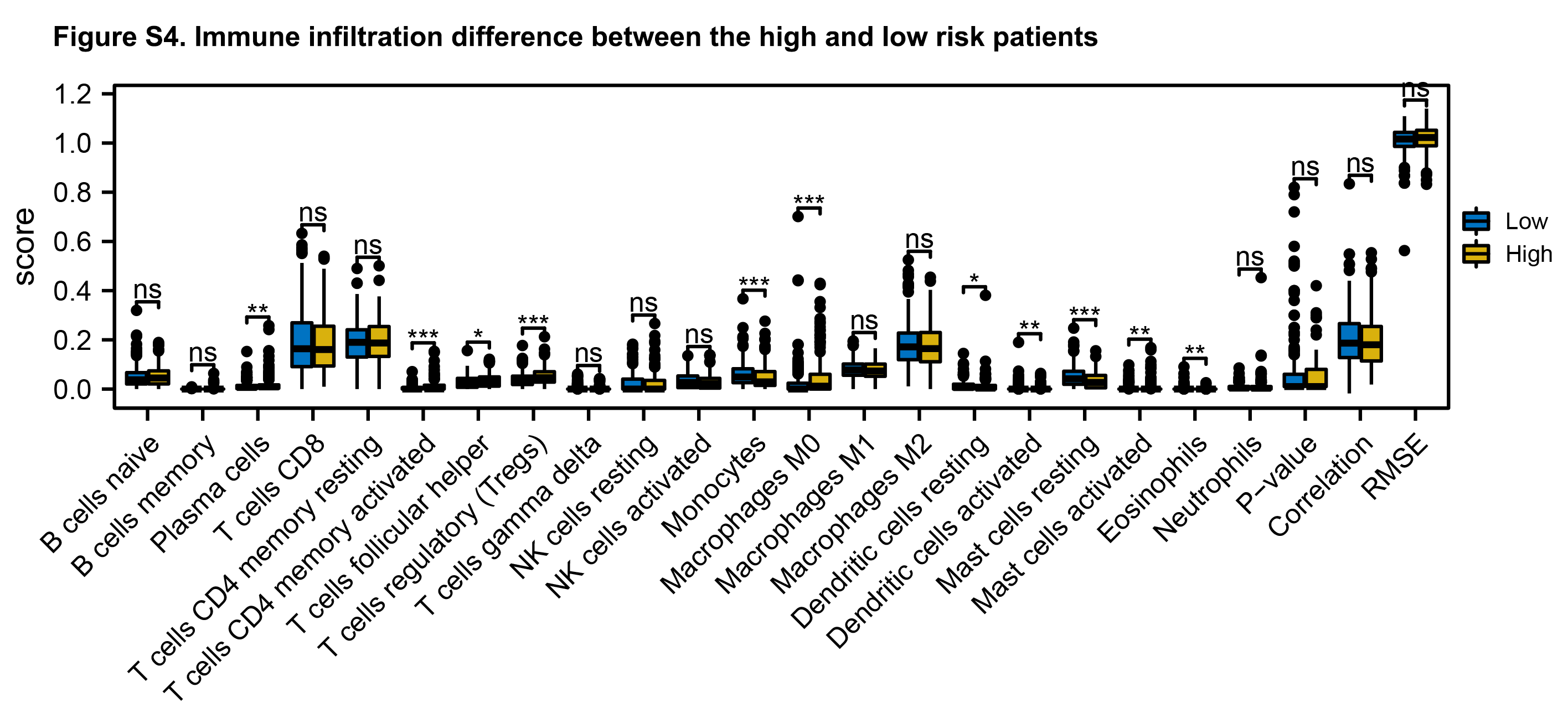

Supplement: Supplementary file 2 [file Image4.TIF]

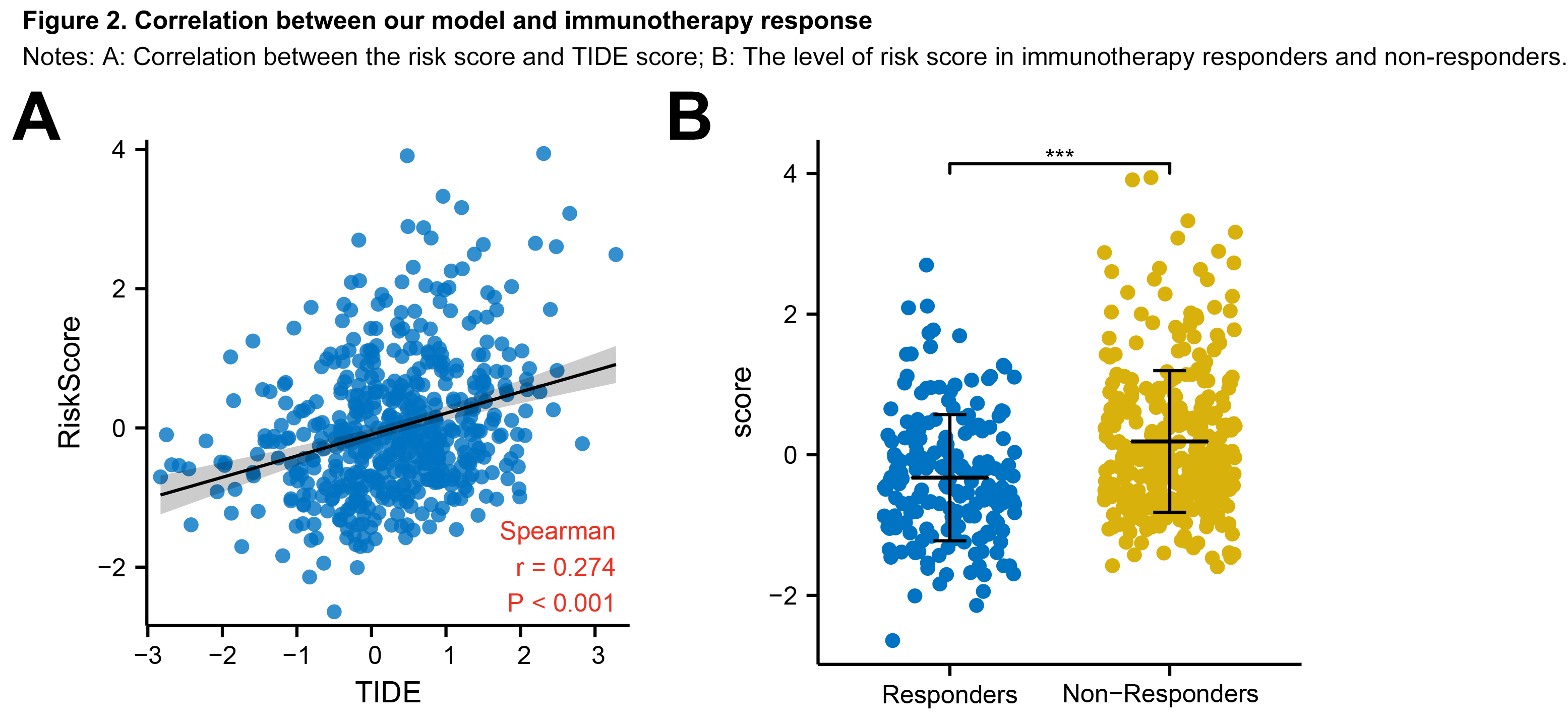

Supplement: Supplementary file 3 [file Image2.TIF]

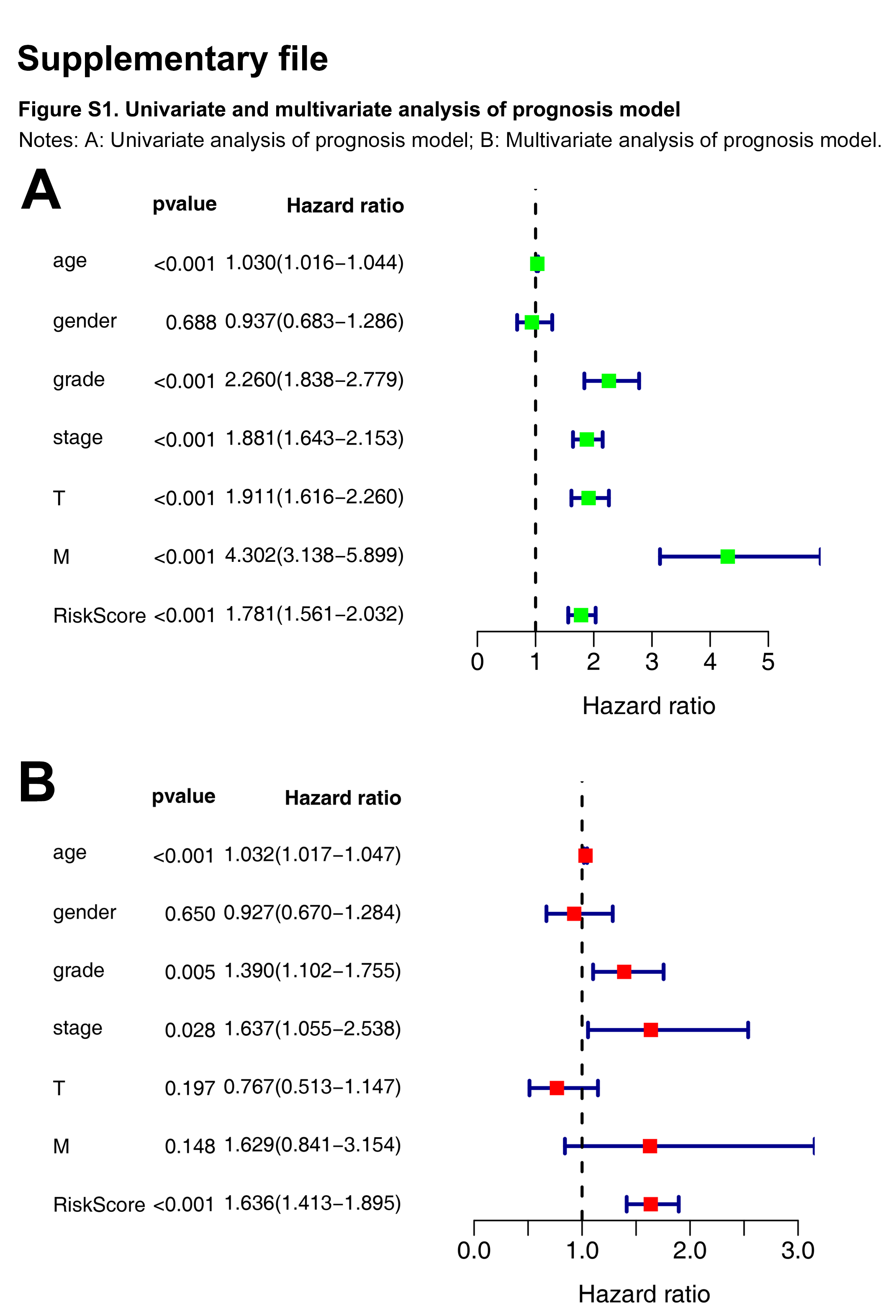

Supplement: Supplementary file 4 [file Image1.TIF]
